# Concurrent three-dimensional conformal radiotherapy and chemotherapy for postoperative recurrence of mediastinal lymph node metastases in patients with esophageal squamous cell carcinoma: a phase 2 single-institution study

**DOI:** 10.1186/1748-717X-9-28

**Published:** 2014-01-19

**Authors:** Dai-yuan Ma, Bang-xian Tan, Mi Liu, Xian-fu Li, Ye-qin Zhou, You Lu

**Affiliations:** 1 Department of Thoracic Oncology, Cancer Center and State Key Laboratory of Biotherapy, West China Hospital, West China Medical School, Sichuan University, 37 Guoxue Lane, Chengdu, P.R. China; 2 Department of Oncology, the First Affiliated Hospital of North Sichuan Medical College, Nanchong 637000, P.R. China

**Keywords:** Esophageal cancer, Lymph node recurrence, Three-dimensional conformal radiotherapy, Concurrent chemotherapy

## Abstract

**Aim:**

The aim of this study was to evaluate the effects of radiotherapy plus concurrent weekly cisplatin chemotherapy on the postoperative recurrence of mediastinal lymph node metastases in esophageal cancer patients.

**Methods:**

Ninety-eight patients were randomly enrolled to receive either three-dimensional conformal radiotherapy alone (group A) or concurrent chemoradiotherapy (group B). A radiation dose of 62–70 Gy/31–35 fractions was delivered to the recurrent tumor. Furthermore, the patients in group B simultaneously received weekly doses of cisplatin (30 mg/m^2^), and the survival outcomes and toxic effects were compared.

**Results:**

The response rate of group B (91.8%) was significantly greater than that of group A (73.5%) (*χ*^2^ = 5.765, *P* = 0.016). The 1- and 3-year survival rates of group B (85.7% and 46.9%, respectively) were also greater than those of group A (69.4% and 28.6%, respectively). However, there were no significant differences in the 5-year survival rates. The numbers of patients who died of distant metastases in groups A and B were 13 (26.5%) and 5 (10.2%), respectively (*χ*^2^ = 4.356, *P* = 0.036). Acute radiation-related esophagitis and granulocytopenia in group B was frequent. However, intergroup differences in terms of late toxicity were not significant.

**Conclusions:**

Three-dimensional conformal radiotherapy (3DCRT) is a practical and feasible technique to treat the recurrence of mediastinal lymph node metastases of postoperative esophageal cancer. In addition, concurrent chemotherapy can increase local tumor control, decrease the distant metastasis rate, and increase the long-term survival rate.

## Introduction

With annual new diagnoses of more than 500,000 patients, esophageal cancer is the eighth most common cancer worldwide [1,2]. It is a highly lethal disease, causing more than 450,000 deaths annually [2]. The incidence of esophageal adenocarcinoma is rapidly rising, whereas that of squamous cell carcinoma remains unvaried in the past few years [1]. Despite accurate preoperative staging, 25% of patients treated with primary surgery have positive microscopic resection margins (R1), and the 5-year survival rate rarely exceeds 40% [3]. Moreover, esophageal squamous cell carcinoma (ESCC) is more deadly in developing countries, such as China, with a 5-year survival rate of only 25% [4]. Although curative esophagectomy with radical lymph node dissection is the primary treatment for early-stage ESCC, the 5-year survival rate remains at 30% [5]. Locoregional recurrence is the major type of failure in ESCC patients following surgery. A previous report has shown that locoregional recurrence was the most common reason for treatment failure, in which mediastinal lymph node metastases accounted for a principal proportion of locoregional relapse in patients with advanced ESCC [6,7]. Clearly, patients with recurrence present with a relatively poorer prognosis [8].

Depending on performance status, a number of advanced ESCC cases have been reported to tolerate salvage treatment [9,10]. In these patients, the potential for a cure existed. Despite no possibility for surgical candidates, radiotherapy, chemotherapy, as well as chemoradiotherapy each have been demonstrated as possible salvage therapies for postoperative locoregional recurrent esophageal carcinoma, with a median overall survival of 7.09–16.0 months [11-13]. In a phase II trial, it has been reported that the combination of radiotherapy (60 Gy/30 fractions) with systematic chemotherapy is a safe and effective salvage option for locorecurrent ESCC [12,14,15]. It is evident that chemotherapy, radiotherapy, and chemoradiotherapy each have displayed definitive responses in a large number of patients with esophageal cancer [16-21]. In spite of all this evidence, there remain controversies regarding the therapeutic strategies and the prognostic risk factors for locoregional recurrence of esophageal cancer.

Three-dimensional conformal radiation therapy (3-DCRT) has been characterized to improve dose distribution, thereby allowing significant increases in the target dose and decreases in lung and heart doses [11,14]. Wu and colleagues have reported refinements of the optimized criteria, including the maximum dose and the important aspects for 3-DCRT [22]. Furthermore, 3-DCRT has been associated with a higher response rate in patients with ESCC, in particular for the tremendous control of mediastinal lymph node metastases [14,20]. ESCC is one of the most common cancers in China [23]. In this study, we prospectively analyzed the efficacy of concurrent 3-DCRT and systematic chemotherapy for treating patients with postoperative recurrence of mediastinal lymph node metastases of ESCC.

## Materials and methods

### Patient eligibility

Between January 2002 and June 2003, 98 patients with histopathologically confirmed advanced locoregional ESCC from the First Hospital affiliated with North Sichuan Medical College, P.R. China, were eligible for inclusion and randomly assigned to the 3-DCRT group (group A) or the 3-DCRT + chemotherapy group (group B) using a random number table. All recruited patients underwent a radical esophagectomy and lymph node dissection for ESCC with a R0 margin, which was defined as the microscopic negative margin of the International Union Against Cancer (UICC) criteria [24]. The locoregional recurrence was ascribed to anastomotic recurrence or locoregional lymph node metastases with a prerequisition of locoregional mediastinal involvement. Subsequently, normal liver, kidney, and bone marrow functions were demonstrated by blood tests. Eligible patients had a good tolerance for radiotherapy or chemotherapy according to the World Health Organization performance status of 0 or 1, without a past history of neoadjuvant or adjuvant chemoradiotherapy following esophagectomy. The candidates who underwent neoadjuvant or adjuvant radiotherapy and/or chemotherapy were excluded from our study. In addition, those with supraclavicular lymph node involvement, only anastomotic stoma recurrence, or hematogenous metastases were ineligible for this study. The basic and clinical characteristics of the studied patients are summarized in Table 1. All patients provided written informed consent. The institutional review board approved the study protocol. The clinical research was registered and approved by the ethics committee of the First Hospital affiliated with North Sichuan Medical College, P.R. China.

**Table 1:** Patient characteristics

**Characteristic**	**Group A**	**Group B**	***χ***^***2***^	***P***
	**(n = 49)**	**(n = 49)**		
**Gender**				
Male	32	35	0.425	0.514
Female	17	14		
**KPS**	80.5 ± 2.13	81.2 ± 1.71	1.794^*^	0.076
**Age (years)**				
Median (range)	54 (38–71)	52 (40–69)	1.515^*^	0.133
**Recurrence time (months)**				
Median (range)	11.4 (6–24)	12.2 (7–23)	1.978^*^	0.051
**Recurrent tumor size** (cm^3^)	9.84 ± 3.32	10.23 ± 4.13	0.515^*^	0.608
**Anatomic recurrence** (number)	11	13	0.221	0.638
**Postoperative pathological stage**				
I + II	30	27	0.377	0.538
III + IV	19	22		
**Recurrence of locoregional lymph node metastases** (number)				
≤2	38	40	0.251	0.616
>2	11	9		

^*^t value.

### Assessment of locoregional mediastinal recurrence

Pretreatment evaluations included complete history, physical examination, imaging evaluation, and laboratory studies. All patients underwent chest X-ray, chest computed tomography (CT), or magnetic resonance imaging (MRI), a barium swallow, electrocardiogram (ECG), esophagoscopy, and ultrasound of the abdomen and pelvis. Mediastinal lymph node metastases were confirmed by the presence of a growing irregular mass by chest CT or MRI. Mediastinal recurrence was characterized as both mediastinal lymph node metastases and anastomotic stoma recurrence of ESCC. Of the 98 eligible patients, 24 were testified as having anastomotic stoma recurrence by histopathological examinations. Alternatively, the following criteria were applied to assess the mediastinal lymph node metastases: the shortest diameter of the suspicious lymph node in the mediastinum was greater than 10 mm; the shortest diameter of the lymph nodes beside the esophagus, at the corner between the esophagus and trachea, or at the cardio-diaphragmatic angle was greater than 5 mm; and several lymph nodes assembled with annular enhancement.

### Radiotherapy and chemotherapy

Four-mm-sectional CT scanning images were obtained and transferred to the Treatment Planning System (TPS) for determination of the target volume and the radiation treatment plan. Gross tumor volume (GTV) included both the mediastinal lymph node region and the recurrent anastomotic region. Subsequently, the clinical tumor volume (CTV) was preliminarily defined as an 8-mm radial margin and a 3-cm vertical margin from the GTV. In addition to this definition, CTV was manually modified to meet the following criteria: 1) To include the nearest adjacent lymphatic drainage regions of the meditational lymph node; 2) To include the 2-cm margin above and below the anastomotic site; 3) To avoid noncancerous organs such as the thoracic stomach, spine, and vessel wall. The planning tumor volume (PTV) was defined as the CTV plus an additional 5-mm margin around the CTV. The organs at risk (OARs), such as the lungs, heart, spinal cord, and thoracic stomach, were also evaluated. A linear 6-MV photon accelerator worked as the X-ray source of radiation. The prescribed dosage for 95% PTV was calculated using 4–6 fields of the coplanar or noncoplanar 3-DCRT plan, which was determined to be 62–70 Gy/31–35 fractions. The radiation plan was divided into two phases. Following a total dose of 46–50 Gy to the CTV, the supplementary plan of the GTV boost was designed to increase the radiation dose for the macroscopic lesions. Conventional radiation plans were designed and performed with 10 Gy in 5 fractions for 1 week (2 Gy/fraction). The doses for the OARs were as follows: maximal dose to the spinal cord, <45 Gy; mean dose to the heart, <40 Gy; percentage of total lung volume receiving ≥20 Gy (V_20_), <30%, dose to the thoracic stomach, V_40_ ≤ 40–50%. Chemotherapy was intravenously administered using cisplatin at a dose of 30 mg per square meter of body-surface area weekly. Chemotherapy was initiated before radiation therapy on day 1 of each week of the therapeutic cycle. In parallel with concurrent radiochemotherapy, the thymic peptide α1 was injected *i.h.* at a dose of 1.6 mg per day for 3 weeks in order to retain systematic immune function. Short-term curative effects were evaluated based on the Response Evaluation Criteria in Solid Tumors (RECIST) [25]. The short-term therapeutic response was assessed based on the complete response (CR) + partial response (PR). Adverse effects were evaluated according to the Common Terminology Criteria for Adverse Events (CTCAE) 3.0 (Grades 1–5). Acute radiation injury was determined according to the Radiation Therapy Oncology Group (RTOG) guidelines (Grades 0–4). Late radiation injury was evaluated based on the RTOG/European Organization for Research and Treatment of Cancer (EORTC) criteria. If therapy was discontinued due to severe toxicity, chemotherapy or radiotherapy was suspended until recovery and the dose was reduced by 25% in the subsequent cycle.

### Follow-up

During the first year after treatment was completed, all eligible patients were followed up every 3 months. During the second year, follow-up took place every 6 months, and then at the end of each year until 5 years following treatment. The response assessment started from the 3rd month after treatment, and patients were followed up for at least 5 years or until death. Late toxic effects, disease progression, and tumor-related death were documented.

### Statistical analyses

Only 2 of the 98 patients were absent from our follow-up assessments. The primary and secondary endpoints were overall survival and severe morbidities of grade 2 or higher, respectively. Overall survival, the primary endpoint, was calculated as the time interval from initiation of treatment to death and was analyzed using the Kaplan–Meier method. Significant differences were analyzed using the log-rank test. The *t* test and *χ*^2^ test were optimized for quantitative and countable data, respectively. All statistical analyses were performed using SPSS 13.0 statistics software (SPSS Inc., Chicago, IL, USA). *P* < 0.05 was considered statistically significant.

## Results

### Response rates

In our study, the “response rate” denoted the ratio of treated patients with CR + PR to the total number of eligible patients. The response rate in group B (45/49, 91.8%) was significantly greater than that in group A (36/49, 73.5%) (*χ*^2^ = 5.765, *P* = 0.016). In addition, group B had a significantly lower presentation of stable disease (SD) and progressive disease (PD) (4/49, 8.2%) as compared with that of group A (13/49, 26.5%) (*χ*^2^ = 5.765, *P* = 0.016).

### Overall survival

**Figure 1:**
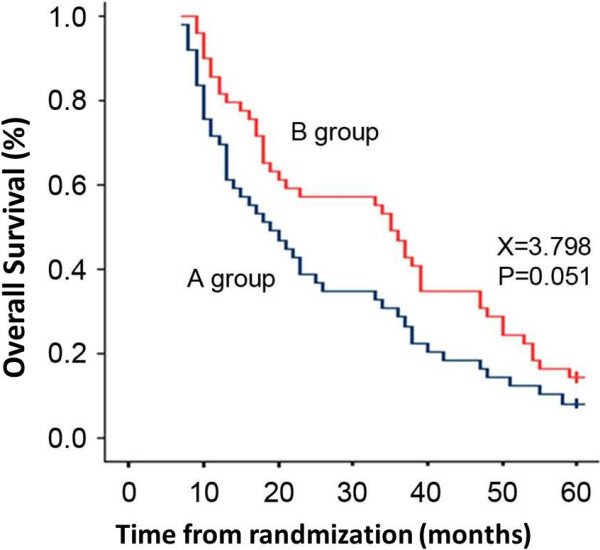
Comparison of the overall survival in both groups.

For surviving patients, the median follow-up period was 60 months (range, 8–63). The intention-to-treat analyses showed a median overall survival of 19 months in group A versus 35 months in group B (*P* = 0.051 by the log-rank test; hazard ratio, 0.76; 95% CI, 28–34) (Figure 1). According to our outcomes, the therapeutic regimen, radiation plus cisplatin-based chemotherapy, had an impressive contribution to the survival benefit in patients with advanced locoregional ESCC (Table 2 and Figure 1). Although there was no remarkable difference in the overall survival rate at five years between both groups (*P* = 0.051), the overall survival rates at 1 year and 3 years in group B were significantly better than those in group A (*P* = 0.032, *P* = 0.038) (Table 2).

**Table 2:** Comparison of survival rates of the two groups

	**1-year SR (%)**	**3-year SR (%)**	**5-year SR (%)**	**MS (Mo; 95% CI)**
Group A	69.4	28.6	8.2	19 (14; 26)
Group B	85.7	46.9	14.3	35 (30; 40)
*χ*^*2*^	4.602	4.298	3.798	
*P*	0.032	0.038	0.051	

*SR* survival rate, *MS* median survival, *Mo* months.

Of the 49 patients in group B who underwent concurrent radiochemotherapy, 5 (10.2%) died from distant metastases of ESCC as compared with 13 out of 49 patients (26.5%) in group A (*χ*^2^ = 4.356, *P* = 0.036). Moreover, the number of patients who died from locoregional progression of recurrent ESCC was 19 in group B versus 22 in group A (*χ*^2^ = 0.676, *P* = 0.410).

### Toxic effect of radiochemotherapy

No life-threatening toxic effects were observed in either group, based on the observation that no radiochemotherapy-related deaths occurred in our study. As shown in Table 3, the adverse effects in the hematological and gastrointestinal systems in group B were obviously more common than in group A. However, there was no significant difference between the incidence of late adverse effects between both groups (Table 3).

**Table 3:** Treatment-related toxic effects

**Group**	**Acute toxicity ≥ grade 2 (*n*)**					**Late toxicity (*n*)**		
	***Esophagitis***	***Leukopenia***	***Nausea and vomiting***	***Diarrhea***	***Hepatic and renal dysfunction***	***Radiation pneumonia***	***Stomal stenosis***	***Heart injury***
A	10	8	5	1	0	1	1	0
B	22	15	14	3	2	4	3	2
*χ*^2^	6.682	2.784	5.290					
*P*	0.01	0.010	0.021	0.358^*^	0.495^*^	0.362^*^	0.242^*^	0.495^*^

^*^Fisher’s exact probability.

### Multivariate analyses for prognostic factors

The prognostic factors for all recruited patients with advanced locoregional ESCC are shown in Table 4. Four risk factors, including GTV of radiation (≥5 cm^3^ versus <5 cm^3^), postoperative T stage (stage I/II versus stage III/IV), locoregional recurrence of lymph node metastases (≥2 versus <1), and chemotherapy treatment (yes versus no) were characterized as significant independent prognostic factors. Patients who had small GTV, early postoperative T stage, small number of locoregional recurrent lymph node metastases, and chemotherapy treatment were likely to have a better prognosis.

**Table 4:** Multivariate analyses for prognostic factors in both groups

	**β**	**Sx**	***χ***^***2***^	***P***
**GTV (<5 cm**^**3**^**vs. ≥5 cm**^**3**^**)**	1.452	0.343	6.673	0.003
**Postoperative T stage (I + II stage vs. advanced stage)**	0.685	0.275	1.843	0.005
**Regional recurrence of lymph node metastases (=1 vs. >1)**	0.021	0.004	1.016	0.002
**Chemotherapy (Yes vs. No)**	1.035	0.395	6.879	0.009

β: regression coefficient; Sx: standard error.

## Discussion

With the increasing incidence of ESCC in Asia, it has been reported that the recurrence rate of patients with ESCC following surgery ranges from 36.1% to 64.2%. [6,20,21]. Overall survival rates at 2 and 5 years are 22% and 11%, respectively, with a median survival time of only 7 months [16,17]. Locoregional recurrence after curative esophagectomy in patients with esophageal cancer remains a challenge to clinical oncologists, regardless of the pathological type. The National Comprehensive Cancer Network (NCCN) guidelines indicate that a subgroup of patients with postoperative locoregional recurrence may be able to tolerate concurrent radiochemotherapy [9,16,26]. The limited survival benefit may arise from the limited competence of the routine radiation technique. With the disadvantage of localization using 2-dimensional radiation fields, the radiation doses for the targeted tumor in patients with locoregional recurrent ESCC could not satisfy the demands of the prescribed doses. Curative full-dose radiation may be sacrificed partially to affect the radiation-related benefits for the therapeutic regimen. In addition, inadequate blood circulation and hypoxic situations following operative implementation render the locoregional site to be insensitive to radiotherapy [27].

An enhancement of virtual simulation is 3-DCRT, in which the radiation beams are shaped to fit the target. When the treatment conforms to the shape of the tumor, the relative toxicity of radiation to the surrounding normal tissues is reduced, allowing a higher dose of radiation to be delivered to the tumor than conventional techniques allow [5,28,29]. With regard to limited survival benefits from irradiation alone, concurrent radiochemotherapy is expected to be another crux of treatment for recurrent ESCC, which depends on the theory that the tumor cell cycle phase is synchronized by chemotherapy, thus enhancing the sensitivity of radiotherapy. Patients with resectable ESCC underwent preoperative neoadjuvant radiochemotherapy and had a favorable outcome in the local control and a remarkable clinical response rate.

Shioyama et al. [18] reported that a better overall survival is obtained for small recurrent lesions, radiation doses more than 50 Gy, and patients with better health scores. In another study, 85% (84/98) of recurrent mediastinal lymph node metastases were located around the superior bronchus, the areas between the vessels of the anterior mediastinum and the back of the tracheae, or the inferior bronchus. Four risk factors, including the GTV of radiation, postoperative T stage, locoregional recurrence of lymph node metastases, and chemotherapy treatment were characterized as significant independent prognostic factors. The discrepancy may result from the eligible criteria for patient recruitment. After effectively protecting the spinal cord, lungs, stomach, and heart from radiation, the target dose was appropriately increased. Concurrent chemotherapy, administered by low-dose cisplatin (30 mg per square meter of body surface), was infused on day 1 of radiotherapy every week. Although acute toxic effects, including esophagitis, nausea, vomiting, and leukopenia, were observed in the study, the therapeutic regimen was completed after the patients received symptomatic treatment. Of note, no life-threatening adverse effects were detected in either group. These results indicated that our regimen for patients with recurrent ESCC is well-tolerable. It was likely that immunotherapy was simultaneously performed with radiochemotherapy to preserve the immune system function that was impaired during the treatment. For locally advanced esophageal cancer, a significant improvement in local control and overall survival was achieved with concurrent chemoradiotherapy as compared with radiotherapy alone [11,14,30]. However, the incidence of local failure was still as high as 44–54%. To improve these results, a phase III trial comparing standard-dose radiotherapy (50.4 Gy) and high-dose radiotherapy (64.8 Gy) concurrently combined with 5-fluorouracil/cisplatin was conducted [27,30]. High-dose irradiation was adopted in our study. The overall survival benefit in both groups in this study were in agreement with previous reports [6,14,21]. The findings may be mainly attributed to the fact that all patients enrolled in this study showed no postoperative positive margins or locoregional lymph nodes metastases. Therefore, the pathological CR + PR corresponded to the long-term survival advantage. Of note, none of the eligible patients underwent postoperative adjuvant chemotherapy or radiotherapy, suggesting that the compliance to chemoradiotherapy was warranted in order to preserve organ function. However, it increased the recurrence risk, so the irradiation doses could be appropriately elevated. In parallel, the total duration of chemotherapy was likely to be shortened using the therapeutic plan with low dose monotherapy. The exposure dose to the OARs was sharply decreased by noncoplanar 3D fields in the head-foot direction. Therefore, the radiation dose was appropriately increased according to the same normal tissue complication probability. A limitation for our study was the relatively small sample size, which may limit the study power.

## Conclusions

In summary, the combined modality of 3-DCRT and chemotherapy was well tolerated compared to radiation alone and yielded superior overall survival rates in patients with postoperative recurrence of mediastinal lymph node metastases of ESCC. Further studies are needed to further optimize this modality to benefit overall survival.

## Abbreviations

3DCRT: Three-dimensional conformal radiotherapy; PTV: Planning tumor volume; CR: Complete response; PR: Partial response; ESCC: Esophageal squamous cancer; UICC: International Union Against Cancer; CT: Computed tomography; MR: Magnetic resonance; TPS: Treatment Planning System; GTV: Gross tumor volume; CTV: Clinical tumor volume; OARs: Organs at risk.

## Competing interests

The authors declare that they have no competing interests.

## Authors’ contributions

YL contribute the primary idea of the study and guide it, D-YM took the head to carry out the scheme and draft the paper. B-xT, ML, X-fL and Y-qZ made substantive intellectual contributions of clinical implement about the study. All authors read and approved the final manuscript.
